# Metabolic imprint induced by seed halo-priming promotes a differential physiological performance in two contrasting quinoa ecotypes

**DOI:** 10.3389/fpls.2022.1034788

**Published:** 2023-02-14

**Authors:** Leonardo Cifuentes, Máximo González, Katherine Pinto-Irish, Rodrigo Álvarez, Teodoro Coba de la Peña, Enrique Ostria-Gallardo, Nicolás Franck, Susana Fischer, Gabriel Barros, Catalina Castro, José Ortiz, Carolina Sanhueza, Néstor Fernández Del-Saz, Luisa Bascunan-Godoy, Patricio A. Castro

**Affiliations:** ^1^ Instituto Forestal (INFOR), Sede Diaguitas, La Serena, Chile; ^2^ Laboratorio de Fisiología Vegetal, Centro de Estudios Avanzados en Zonas Áridas (CEAZA), La Serena, Chile; ^3^ Centro de Estudios en Zonas Áridas (CEZA), Facultad de Ciencias Agronómicas, Universidad de Chile, Coquimbo, Chile; ^4^ Laboratorio de Fisiología Vegetal, Departamento de Producción vegetal Facultad de Agronomía, Universidad de Concepción, Concepción, Chile; ^5^ Laboratorio de Fisiología Vegetal, Departamento de Botánica, Facultad de Ciencias Naturales y Oceanográficas, Universidad de Concepción, Concepción, Chile; ^6^ Departamento de Fisiología, Facultad de Ciencias Biológicas, Universidad de Concepción, Concepción, Chile

**Keywords:** halophyte, preconditioning, salt-tolerant, photosynthetic performance, memory, metabolomic, *Chenopodium quinoa*

## Abstract

“Memory imprint” refers to the process when prior exposure to stress prepares the plant for subsequent stress episodes. Seed priming is a strategy to change the performance of seedlings to cope with stress; however, mechanisms associated with the metabolic response are fragmentary. Salinity is one of the major abiotic stresses that affect crop production in arid and semiarid areas. *Chenopodium quinoa* Willd. (Amaranthaceae) is a promising crop to sustain food security and possesses a wide genetic diversity of salinity tolerance. To elucidate if the metabolic memory induced by seed halo-priming (HP) differs among contrasting saline tolerance plants, seeds of two ecotypes of Quinoa (Socaire from Atacama Salar, and BO78 from Chilean Coastal/lowlands) were treated with a saline solution and then germinated and grown under different saline conditions. The seed HP showed a more positive impact on the sensitive ecotype during germination and promoted changes in the metabolomic profile in both ecotypes, including a reduction in carbohydrates (starch) and organic acids (citric and succinic acid), and an increase in antioxidants (ascorbic acid and α-tocopherol) and related metabolites. These changes were linked to a further reduced level of oxidative markers (methionine sulfoxide and malondialdehyde), allowing improvements in the energy use in photosystem II under saline conditions in the salt-sensitive ecotype. In view of these results, we conclude that seed HP prompts a “metabolic imprint” related to ROS scavenger at the thylakoid level, improving further the physiological performance of the most sensitive ecotype.

## Introduction

Plants modulate their physiological responses in order to acclimate to stressful conditions in their local environment. Priming has emerged as a tool that may benefit plant physiology and metabolism under stress conditions (Ashraf and Foolad, 2005; Paparella et al., 2015). The term “stress imprint” can be defined as the priming or hardening process in which prior exposure to environmental stress increases plant resistance to future exposures (Bruce et al., 2007). Multiple examples of imprint have been observed in higher plant species in response to drought, salinity, and high and low temperatures, among others (Ashraf and Foolad, 2005; Paparella et al., 2015; Ibrahim, 2016). In seeds of several species, priming improves germination and uniform emergencies and improves seedling’s vigor, establishment, and growth (Kaya et al., 2006; Dezfuli et al., 2008; Bakht et al., 2011; Manonmani et al., 2014; Raj and Raj, 2019). Mechanisms associated with priming may occur at different levels, including epigenetic (e.g., by DNA and histone modification), transcriptional (e.g., changes in the abundance of transcripts and transcription factors), protein level, and by modulating enzyme activities (Schwachtje et al., 2019). However, the priming-related changes in the metabolism remain largely unexplored, although it is highly compromised during stress (Schwachtje et al., 2019).

Salinity is one of the most brutal environmental factors limiting crop productivity (Shrivastava and Kumar, 2015), with germination being one of the most critical salt-sensitive periods (Jacobsen et al., 1999; Maleki et al., 2018). *Chenopodium quinoa* (Willd.) is an annual crop native from the Andean region and has been nominated as one of the crops that could contribute to global food security during the next century (FAO, 2011; Ruiz-Carrasco et al., 2011; Adolf et al., 2013). Even though quinoa is considered a facultative halophyte (Biondi et al., 2015), there is a great gradient in saline tolerance according to its origin (Delatorre-Herrera and Pinto, 2009; Razzaghi et al., 2011). Altiplano and Lowlands are two representative quinoa ecotypes with contrasting salinity resilience (Jacobsen and Stølen, 1993; Delatorre-Herrera and Pinto, 2009; Ruiz-Carrasco et al., 2011). Recent reports indicate that different seed priming treatments (chemical priming, osmopriming, saponins, etc.), including halo-priming (pretreatment with a low level of salt), improved germination under salt stress conditions in quinoa (Daur, 2018; Moreno et al., 2018) and mitigated the negative effects of salt stress on growing (Yang et al., 2018).

It is reported that quinoa plants reconfigure their metabolism under salt stress conditions. Comparative studies between tolerant and sensitive genotypes reveal enrichment of diverse pathways including photosynthesis-related, phenylpropanoid biosynthesis, tyrosine metabolism, and ROS scavenging pathways in tolerant salt-sensitive genotypes (Shi and Gu, 2020; Derbali et al., 2021). Interestingly, the stress memory is not the simple repetitious activation of the same strategies of the original stress (Liu H et al., 2022), and the metabolic pathways induced by seed HP in quinoa and their impact on plant performance are far from being clarified. Moreover, as has been demonstrated in rice, the extent of the effect of memory could be different between sensitive and tolerant genotypes (do Amaral et al., 2020).

In this work, we wonder: Which are the metabolic pathways induced by salinity? Could seed halo-priming be involved in the induction of a metabolic memory that can enhance salt tolerance during growing? What is the link of this metabolic imprint with plant physiological performance? Could this response be different depending on the saline tolerance of the ecotype? The identification of metabolic pathways involved in the imprint of stress memory may provide potential keys for global food production in a scenario of climatic change.

## Materials and methods

### Plant material

Seeds of two Chilean ecotypes of *C. quinoa* (Willd.) from contrasting agro-ecological origins were used in the experiments: the Socaire ecotype from the Chilean Altiplano (Socaire, 23°35’31.58” S, 67°53’17.69” W) and the coastal BO78 ecotype (Temuco, 39°10’ S, 72°68’ W). Seeds were obtained from the National Seed Bank of Chile managed by the Genetic Resources section of the National Institute of Agriculture Research (http://163.247.128.32/gringlobal/search.aspx, INIA-Intihuasi Vicuña, Chile).

### Seed sensitivity test to salt

Considering that salinity tolerance in quinoa does not always correlate with geographic distribution, to reveal a differential saline tolerance on germination traits between Socaire and BO78 ecotypes, we evaluated the effect of different salt concentrations over seed germination. Seeds were incubated at 0, 200, 250, 300, 350, and 400 mM NaCl for 48 h at 24°C in the dark. Fifty seeds were used for each treatment, and experiments were performed in triplicate. The final germination percentage, mean germination time, and mean velocity germination were recorded every 4 h up to 48 h after seed imbibition following Scott et al. (1984) and Ranal and Santana (2006). Germination was considered complete when the radicle emerged from the seed.

### Effect of saline shock on net photosynthesis in two contrasting ecotypes

In order to evaluate the performance of Socaire and BO78 ecotypes under salt stress during the vegetative stage, plants were exposed to a saline shock treatment under greenhouse conditions. Seeds of Socaire and BO78 were sown directly in 5 kg of dry soil, in 10-L pots (22 cm tall by 28 cm diameter), containing a mixture of sand and peat in a 2:1 ratio and fertilized with 4 g of 6 M Basacote Plus Compo Expert (16% N, 3.5% P, 10% K, 1.2% Mg, 5% S, and micronutrients). The environmental conditions were as follows: 1,200 μmol m^−2^ s^−1^ PAR at noon (natural light); maximum and minimum temperatures (daily ranges) of 23°C and 17°C, respectively; 12-h day length; and 80% relative humidity. Seedlings were irrigated with distilled water at field capacity, and the water content of each pot was monitored every 3 days by weight, and additional irrigation was applied for moisture fulfillment. At the stage of six pairs of true leaves (24 days after germination), seedlings (*n* = 12) were subjected to a saline shock by unique irrigation with 400 mM NaCl solution (~ the salinity of sea water, electrical conductivity of 40 dS/m; Adolf et al., 2013), while control plants were maintained irrigated with distilled water. After the application of the saline shock treatment, plants under control and salt stresses were irrigated every 2 days with distilled water. The effects of salinity stress and recovery were determined by evaluating changes in net photosynthetic rate (*P_n_*) 10 days after the application of the salt treatment. Leaf photosynthetic rate (*P_n_*) was measured in fully expanded leaves during mid-morning (between 11:00 a.m. and 13:00 p.m.) with a gas exchange system (Li-6400, Li-Cor Inc., Nebraska, USA) equipped with a light source (6200-02B LED, Li-Cor). Environmental conditions in the leaf chamber were as follows: photosynthetically active photon flux density = 1500 µmol photon m^−2^ s^−1^ and CO_2_ concentration (Ca) = 400 µmol mol^−1^. The experiment was performed in a completely randomized design. Plants from pots in border rows of the experimental design were discarded in order to avoid the border effect.

### Effect of halo-priming on seed germination

In order to choose the conditions for halo-priming (HP), seeds of each ecotype were embedded at different times and NaCl concentrations in the dark at room temperature. Considering the improvement of BO78 germination, we selected for HP the solution of 68 mM NaCl by 12 h (**Supplementary Figure S1**). Then, for all experiments, seeds were embedded under those conditions and subsequently washed twice with distilled water and then surface-dried with adsorbent paper at room temperature for 12 h before establishing the germination assays. Unprimed (UP) seeds were not treated with the priming solution. Germination experiments were performed in a completely randomized design with three factors under study: Ecotypes (E), Priming (P), and Saline treatment (S). Haloprimed (HP) and unprimed (UP) seeds of both ecotypes were submitted to control (C, distilled water) or saline (S, 300 mM NaCl) treatment. This concentration reduced germination-related parameters in both ecotypes and has been widely used to compare salt tolerance among quinoa genotypes (Ruiz et al., 2017). Germination was carried out as previously described, and the germination parameters (final germination percentage, mean germination time, and mean velocity germination) were recorded after 48 h in both ecotypes to each treatment (Unprimed Control: UP-C; Haloprimed Control: HP-C; Unprimed Salt: UP-S; Haloprimed Salt: HP-S).

### Effect of seed priming on seedling physiological performance under salt treatment

To study the establishment of juvenile plants in pots, UP and HP seeds of Socaire and BO78 were sown directly in soil (*n* = 12; 96 plants in total). Four seeds per treatment were sown in order to obtain one established seedling per pot. The environmental conditions of the greenhouse were as follows: 1,200 μmol m^−2^ s^−1^ PAR at noon (natural light); maximum and minimum temperatures (daily ranges) of 23°C and 17°C, respectively; 12-h day length; and 80% relative humidity. From seed imbibition to seedling growth, two irrigation treatments were applied: Control (C) by irrigation with distilled water and Saline (S) with increased soil salinization by weekly irrigation of 100 mM NaCl. Pots were irrigated to 90% of soil water-holding capacity to minimize NaCl losses from soil. The experiment was run in a completely randomized design and supplementary plants were used in order to prevent the border effect.

At 24 days after germination (at the stage of six pairs of true leaves), parameters from chlorophyll *a* fluorescence were recorded. Plants were collected for biochemical analysis (*n* = 6) including determination of chlorophyll content, lipid peroxidation, starch, soluble sugars, proline, and metabolomic profile.

### Chlorophyll *a* fluorescence

Chlorophyll *a* fluorescence parameters were obtained by an FMS 2 portable fluorometer (Hansatech Instruments, UK) as described by Bascuñán-Godoy et al. (2012); Bascuñán-Godoy et al. (2015). Fully expanded leaves of plants from each ecotype and treatment (*n* = 6) were dark-adapted at room temperature for 30 min prior to measurement. The intensity of actinic light for measurements was 1,200 mmol photons m^−2^ s^−1^. The following parameters were recorded: maximum photochemical efficiency of photosystem II (Fv/Fm), effective quantum yield (ΦPSII), and thermal dissipation measured as non-photochemical quenching (NPQ) (Maxwell and Johnson, 2000).

### Lipid peroxidation, total soluble sugars, starch, and proline content

Fully expanded leaves of plants from each ecotype and treatment (*n* = 6) were used for the spectrophotometric analysis of metabolites. Fresh leaf tissue (100 mg) was collected, flash-frozen in liquid nitrogen, and stored at −80°C to posterior analysis.

The lipid peroxidation in leaves was assayed using the thiobarbituric acid method according to Ortega-Villasante et al. (2005). This test determines malondialdehyde (MDA) as an end product of the thiobarbituric acid reaction. Total soluble sugars (TSS) and starch were determined according to McCready et al. (1950) and Rose et al. (1991), respectively. Chlorophyll extraction was performed according to Mackinney (1941), while its concentrations (*a* and *b*) were determined according to Lichtenthaler and Buschmann (2001). Proline (Pro) was determined at 520 nm using the rapid method developed by Bates et al. (1973).

### Metabolome profiling

For metabolome profiling, leaves from plants subjected to different treatments were frozen and then freeze-dried. Metabolome profiling analysis was performed at the West Coast Metabolomics Center (UC Davis, Davis, CA, USA), according to the methodology described in Botanga et al. (2012). Raw data were normalized, filtered, and analyzed using the MetaboAnalyst 3.0 webserver (Xia et al., 2015).

### Statistical analysis

A two-way analysis of variance (ANOVA) (*p* < 0.05) was conducted to determine the effects of Ecotypes (E, Socaire and BO78) and salinity (S, 0 to 400 mM) on germination parameters. Photosynthetic data were analyzed using repeated measures ANOVA (*p* < 0.05). A three-way ANOVA was conducted in order to determine the effects of Ecotypes (Socaire and BO78), Saline treatment (C or S), and Priming (UP or HP) on physiological parameters and metabolites. Tukey HSD test (level of significance *p* < 0.05) was used as a *post-hoc* test, in order to explore evidence of significant differences. All analyses were performed with the STATISTICA v7.0 software package (StatSoft Inc., Tulsa, OK, USA).

Heatmap plots (to visualize metabolites profiles) were performed using the heatmap R package. Data were scaled by *Z*-score to capture metabolites with similar behavior. Clustering of metabolites was performed using the default pheatmap Ward method. Metabolite pathway information was obtained from KEGG API (https://www.kegg.jp/kegg/rest/keggapi.html) using a Shell script. For metabolome profiling analysis, a principal component analysis (PCA) and hierarchical cluster analysis using Ward clustering algorithm and Euclidian distance were performed to identify relations among variables; normalized data corresponding to different treatments were analyzed by three-way ANOVA.

Statistical interpretations of data were based on the complex interactions that nest the simple effect and independent effects that are not included in an interaction.

## Results

### Effect of salinity on germination of two quinoa ecotypes

**Figure 1:**
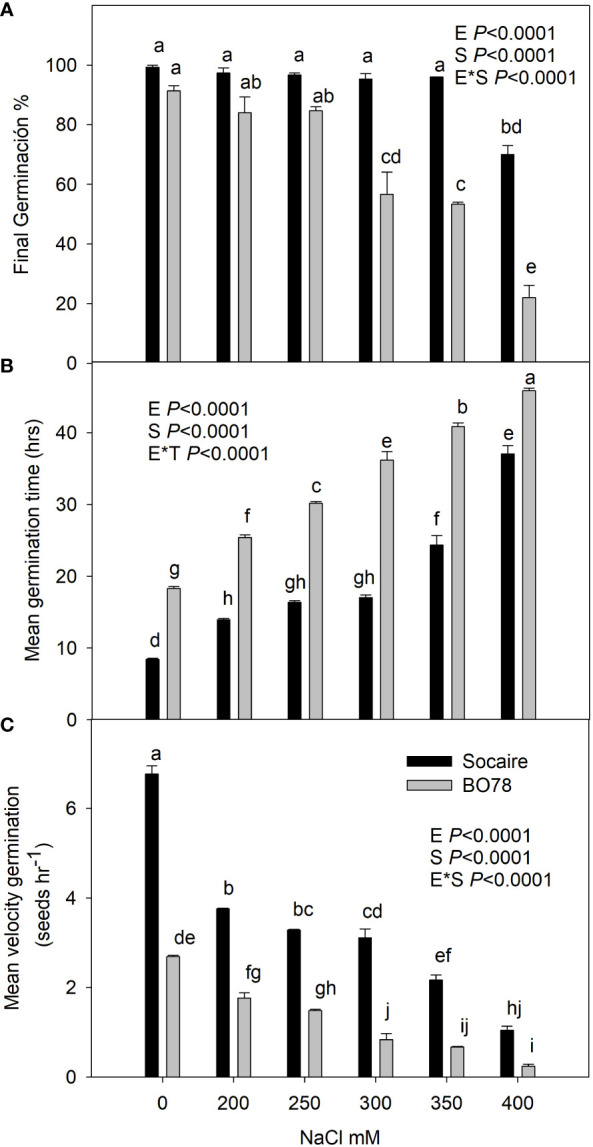
Salinity effect on germination percentage **(A)**, mean germination time **(B)**, and mean velocity germination **(C)** of Socaire and BO78 ecotypes of quinoa seeds. Seeds were germinated under 0, 200, 250, 300, 350, and 400 mM of NaCl. Error bars show mean ± SE (*n* = 12). Different letters indicate significant differences between ecotypes (E) and salinity treatments (T) using two-way ANOVA. Tukey HSD was used as a *post-hoc* test (*p* < 0.05).

In order to verify a differential physiological salt tolerance between the two quinoa ecotypes from contrasting origins, a comparative analysis of germination capacity was performed (**Figure 1**). A statistically significant interaction between plant ecotype (E) and treatment (S) was observed in all the parameters related to germination studied (*p* < 0.0001). After 48 h from seed imbibition, the two ecotypes displayed similar final germination percentages in control and under 200 and 250 mM NaCl with values higher than 85% (**Figure 1A**). Germination values of Socaire were maintained close to 100% in the presence of up to 350 mM NaCl, and only a significant decrease was observed at 400 mM NaCl (70% of germination). Contrastingly, BO78 seeds displayed significant decreases in germination at 300 and 350 mM NaCl (56% of GP), and at 400 mM of NaCl, only 22% of final germination was reached.

The study of mean germination time indicates that Socaire takes 10 h for germination (**Figure 1B**) while BO78 takes 20 h. Additionally, the mean velocity of germination indicates that Socaire germinates 2.5 times faster than BO78 under control conditions (**Figure 1C**). A significant increase in the time for germination and a decrease in velocity was observed in both ecotypes in the presence of NaCl from 200 to 400 mM. A stronger change in those parameter values was observed through all concentrations studied in BO78 than in Socaire (**Figure 1B**).

### Effect of salinity shock on the photosynthetic performance of both quinoa ecotypes

**Figure 2:**
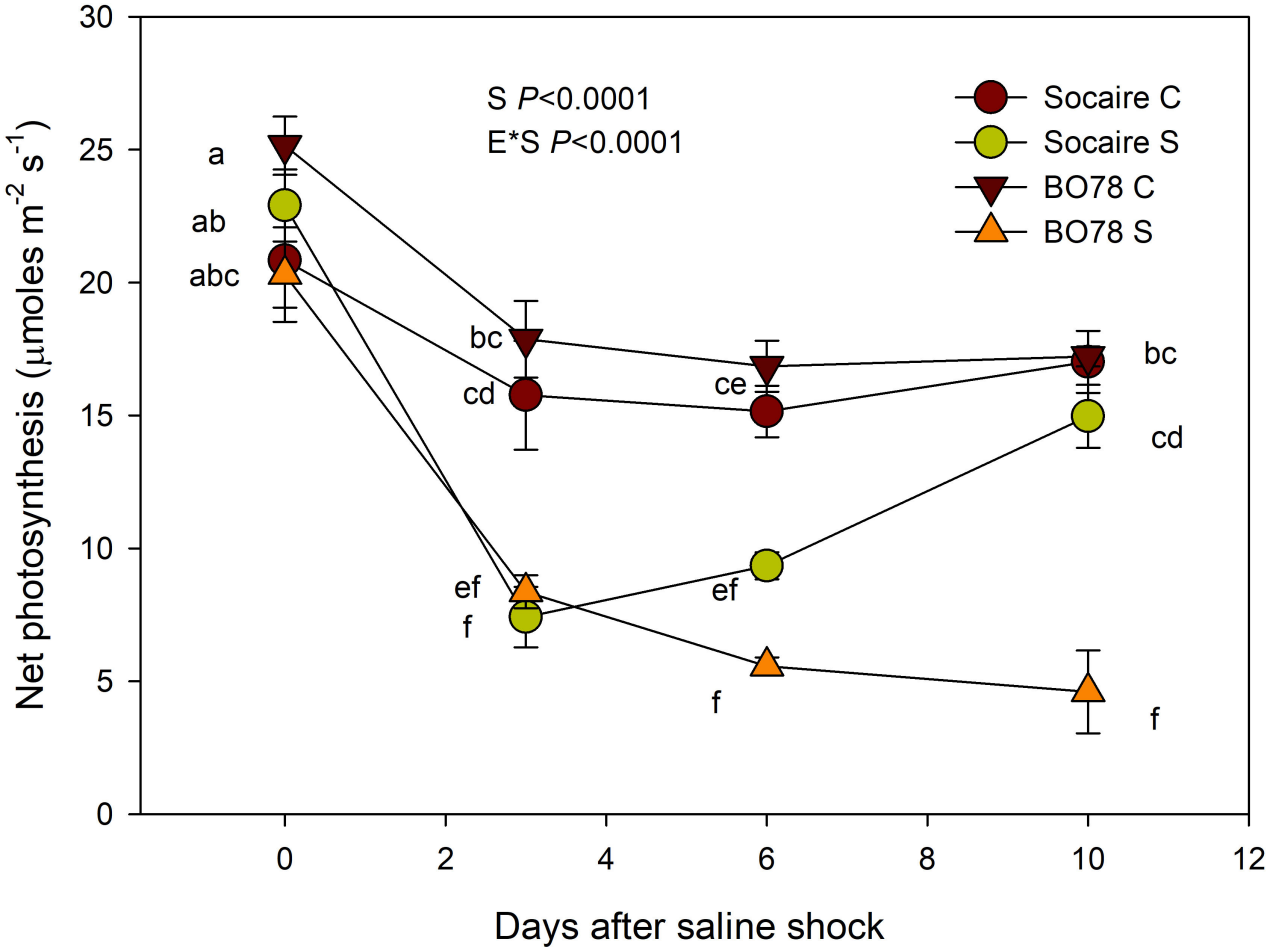
Salinity effect on net photosynthesis (Pn) of Socaire and BO78 ecotypes of quinoa. Plants grown under control conditions (C, 0 mM NaCl) were exposed to salinity shock (S, 400 mM NaCl) and recovered by 10 days. Error bars show mean ± SE (n = 12). Different letters indicate significant differences between ecotypes (E) and salinity treatments (T) using repeated measures ANOVA (*p* < 0.05).

Additionally, to test the effect of salinity on germination parameters, we compared the ability to recover the net photosynthetic rate (*P_n_*) in seedlings submitted to salinity shock (400 mM NaCl). Similarly to germination parameters, we found the interaction E × S (*p* < 0.0001). Prior to the application of saline shock (control conditions, day 0), all the plant groups exhibited similar *P_n_* values **Figure 2** (Net Photosynthesis) Furthermore, a similar and significant decline of about 50% of *P_n_* values was observed in both quinoa ecotypes after 3 days from the salinity shock (**Figure 1**). Interestingly, 10 days after the salinity shock treatment, it was observed that Socaire plants were able to recover *P_n_* values similar to those observed in control plants, while BO78 plants were unable to recover *P_n_*.

### Effect of seed halo-priming on germination

**Figure 3:**
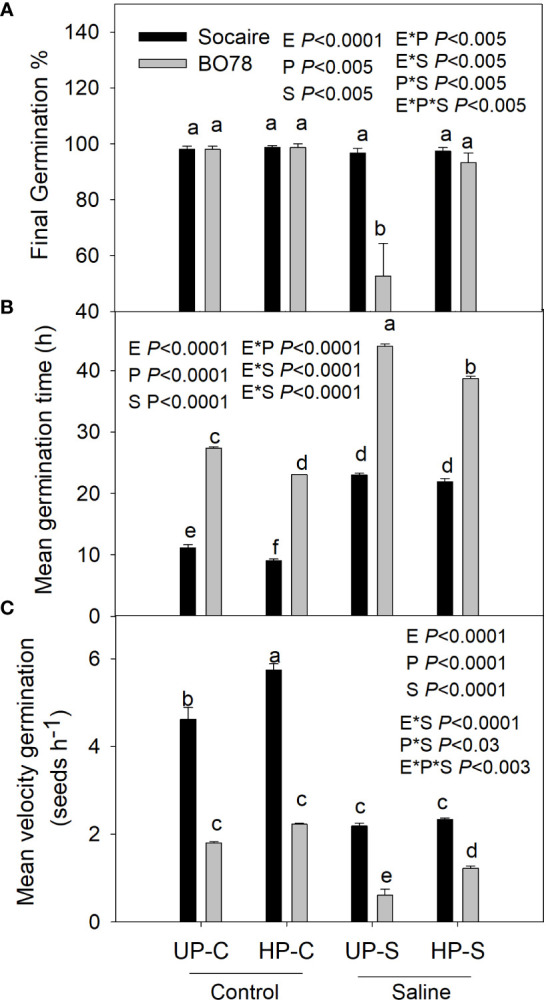
Salinity and priming effect on germination percentage **(A)**, mean germination time **(B)**, and mean velocity germination **(C)** of Socaire and BO78 ecotypes of quinoa seeds. Error bars show mean ± SE (*n* = 12). Different letters indicate significant differences between ecotypes (E, Socaire or BO78), priming (P, UP or HP), and treatment (T, C, or S) using three-way ANOVA. Tukey HSD was used as a post-hoc test (*p* < 0.05).

Once the differential salinity tolerance of the ecotypes studied was verified, halo-priming (HP) studies were carried out. All the parameters related to germination were affected by HP (**Figure 3**), and the final germination percentage and mean velocity of germination were dependent on the interaction among E × S × P (*p* < 0.005).

The final germination percentage of the Socaire ecotype was not reduced by salinity, while in BO78, it decreased to 50% (UP-S treatment) (**Figure 3A**). Remarkably, seed HP reverted the effect of salinity in BO78, reaching 100% of germination.

Salinity treatment prolonged the mean germination time (**Figure 3B**), reducing the speed of germination in both ecotypes (**Figure 3C**). Seed HP significantly reduced germination time and increased the speed of germination under control conditions in Socaire and in both conditions (control and saline) in the most sensitive genotype.

### Effect of seed halo-priming on “chlorophyll *a*” fluorescence parameters in juvenile plants of both ecotypes

**Figure 4:**
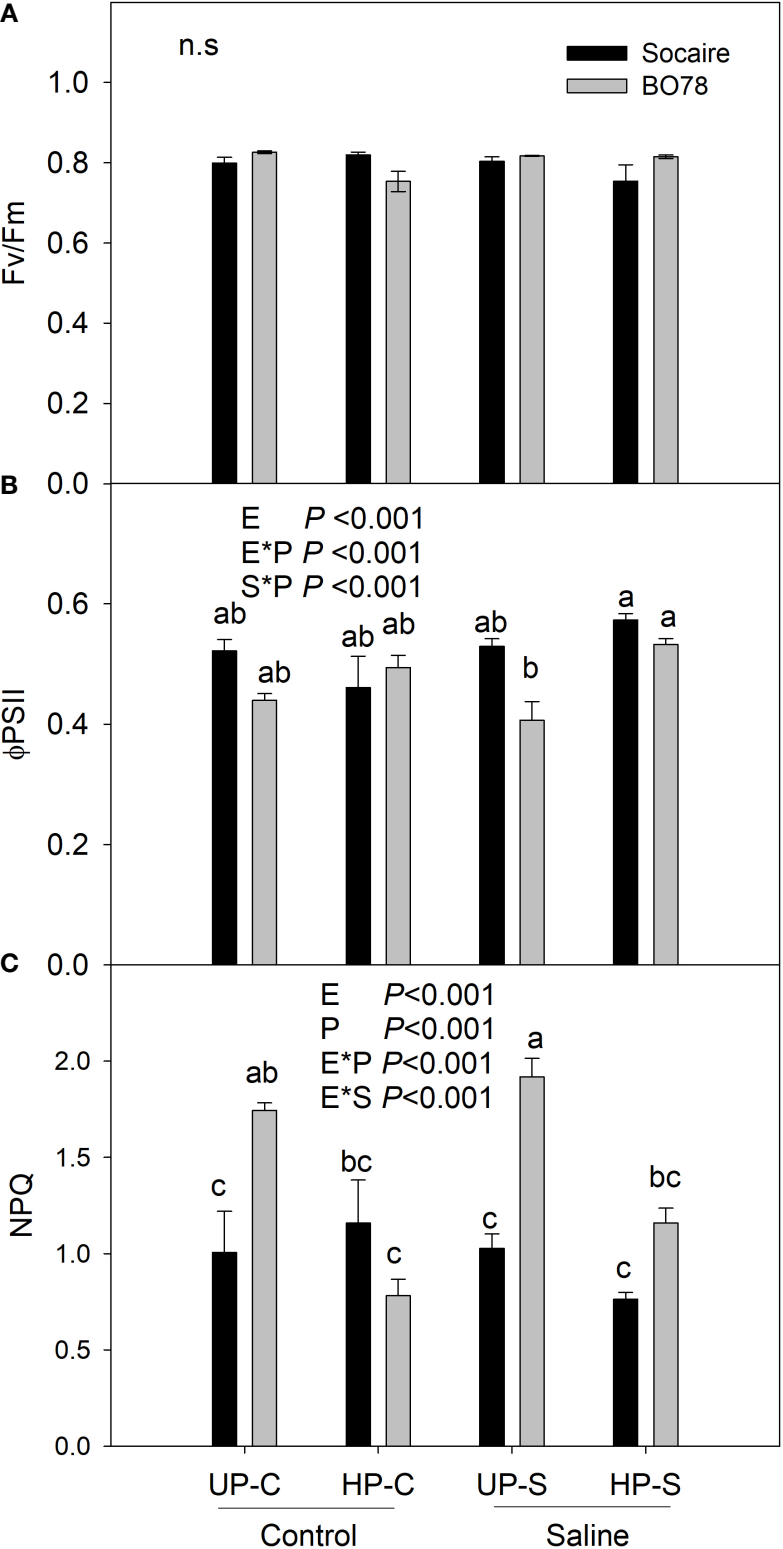
Salinity and priming effect on the chlorophyll *a* fluorescence parameters of Socaire and BO78 ecotypes. Haloprimed and unprimed seeds of both ecotypes were germinated and grown under control and salinity conditions. At 20 days after germination, chlorophyll a fluorescence parameters were registered: Fv/Fm, maximum efficiency of the PSII **(A)**, FPSII, effective quantum yield **(B)**, and NPQ, non-photochemical quenching **(C)**. Error bars show mean ± SE (n = 6). Letters indicate significant differences between ecotypes (E, Socaire or BO78), priming (P, UP or HP), and treatment (C or S) using threeway ANOVA. Tukey HSD was used as a post-hoc test (*p* < 0.05).

Seedling of both *C. quinoa* ecotypes showed similar values of the maximal fluorescence of PSII (Fv/Fm) that were maintained at about 0.8 through the treatments **Figure 4** (Chlorphyll fluorescence). The quantum yield of photosynthesis (ΦPSII) was dependent on the interactions between E × P (*p* < 0.001) and S × P (*p* < 0.001) (**Figure 4B**). Ecotypes presented similar levels of ΦPSII at control conditions (UP-C), and salinity treatment negatively affected only BO78. Seed HP enhanced 30% ΦPSII value in this ecotype (BO78) under saline conditions (HP-S), while no effect was observed in Socaire.

NPQ was dependent on the interaction of E × S (*p* < 0.001) and E × P (*p* < 0.001). Saline treatment tended to increase NPQ in BO78 but not in Socaire (**Figure 4C**). Seed HP significantly reduced NPQ values in BO78 at both control and saline conditions (HP-C and HP-S), to similar levels to those observed in Socaire (**Figure 4C**). Interestingly, this decrease in NPQ values in BO78 HP-S plants (reduction of 40%) was concomitant with the enhanced ΦPSII values.

### Effect of seed HP and salinity treatment on lipid peroxidation, carbohydrates, chlorophylls, and proline

**Figure 5:**
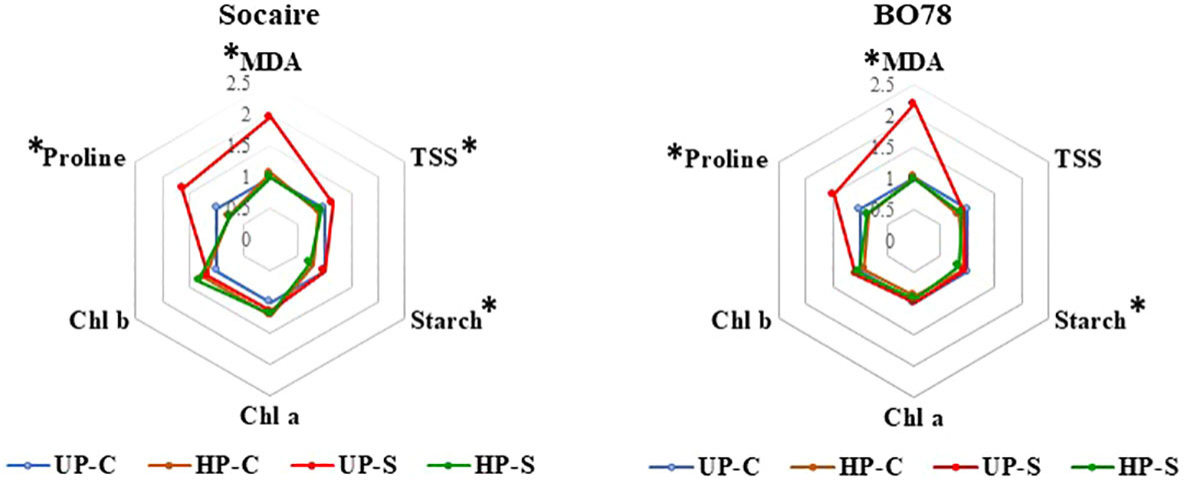
Salinity and priming effect on metabolites in Socaire and BO78 ecotypes of quinoa. Radar chart shows relative changes in lipid peroxidation (measured as MDA levels), total soluble sugars (TSS), starch, chlorophylls (Chl *a* and *b*), and proline. Relative changes were calculated as the ratio of the content of each metabolite to UP-C samples, for each ecotype. Asterisks indicate significant differences among treatments.

The levels were calculated as the ratio of each value to the Control (UP-C) treatment (**Figure 5**). Original data of measurements and concentrations at the different conditions are summarized in **Supplementary Table 1**.

MDA (a product of membrane lipid peroxidation) level was dependent on the interaction of E × S × P (*p* < 0.009, **Supplementary Table 1**). MDA increased about two times in both ecotypes under saline treatments, reaching the highest value in BO78 UP-S. Seed HP reduced the content of MDA to similar levels to control conditions (UP-C) (**Figure 5**).

Total soluble sugar (TSS) amount was dependent on the interaction of E × S × P (*p* < 0.005, **Supplementary Table 1**). Salinity (UP-S treatment) induced a significant increase in the content of TSS in Socaire, which was not observed in BO78 (**Figure 5**). Halo-priming induced a significant decrease in TSS, which was most notorious in Socaire HP-S (**Supplementary Table 1**).

BO78 stored an 18% higher amount of starch than Socaire plants (**Supplementary Table 1**) and HP reduced the starch in both ecotypes in control and saline conditions (*p* < 0.001). No interaction among factors was observed for leaf starch amount.

Chlorophyll levels were dependent on the interaction E × P (*p* = 0.01). Both Chl *a* and *b* contents were significantly higher in BO78 than in Socaire, and tended to increase by HP effect but only in Socaire (**Figure 5** and **Supplementary Table 1**).

Proline was dependent on the interaction of P × S (*p* = 0.008). A significant reduction in proline content was observed in Socaire and BO78 plants submitted to saline treatment because of the effect of HP (**Figure 5**).

### Metabolome profiling

Metabolome profiling was performed to gain insight into the metabolic changes in Socaire and BO78 ecotypes upon seed halo-priming and subsequent growing at control or saline treatment. A total of 371 metabolites were detected, 161 of which were identified by assignment to a PubChem or KEGG code.

**Figure 6:**
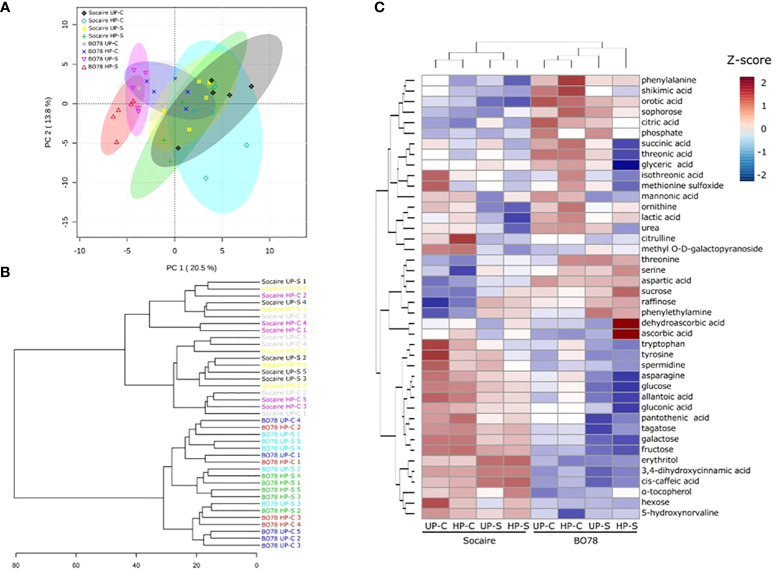
Principal component analysis (PCA) **(A)**, hierarchical clustering dendrogram **(B)**, and heatmap for metabolome profiling data **(C)**. The PCA, hierarchical clustering dendrogram, and clustered heatmap show the most changing metabolites (42 metabolites) under the different conditions (UP-C, HP-C, UP-S, and HP-S) in both ecotypes. Heatmap scale bar shows standardized metabolite values color-coded from low (blue) to high (red).

A PCA was applied to the different relative parameters to identify those that accounted for the major component of variability. The PCA revealed two components that accounted for 34.3% of the total variance. On one hand, PC1 (associated with ecotype) accounted for 20.5% of the variance, while the second component PC2 (associated with halo-priming and salt treatments) accounted for 13.8% of the total variance (**Figure 6**).

In addition, hierarchical cluster analysis of data showed that all biological replicates clustered into two clades, which matched with both quinoa ecotypes (**Figure 6B**). Clade I is composed of all Socaire replicates; no match between clades and treatments was observed (**Figure 6B**). Clade II is composed of replicates of the BO78 ecotype; interestingly, all replicates of BO78 priming plants and submitted to salinity conditions were grouped in a single cluster (**Figure 6B**).

According to the metabolomic analysis, 99 metabolites were differentially accumulated among ecotypes, salinity and halo-priming, or interactions. Of them, 42 metabolites were identified in PubChem or KEGG database and 57 were unknown. The identified metabolites belong to different pathways, including the Shikimic acid pathway, amino acids, TCA cycle, ornithine cycle, sugar metabolism, fatty acid metabolism, purine metabolism, and ascorbic acid pathway (detailed in **Figure 6C**). The *p*-values of the effect of the different factors on these 42 metabolites are included in **Table 1**. Significant changes in metabolites are highlighted in red and bold, while the independent effect factors (not included in any further interaction) were underlined. From the identified metabolites, it was observed that 30 displayed significant differences between ecotypes, 31 were modified in response to salinity, and 10 changed significantly by seed HP (**Figure 6C** and **Table 1**).

**Table 1:** *P*-values for the effects of Ecotype (E), Salt treatment (S), Priming (P), and their interactions, determined by three-way ANOVA analysis, on metabolites during the vegetative stage.

	Ecotype	Salt	Priming	E × S	E × P	P × S	E × S × P
**Glucose**	**0.00**	**0.00**	0.71	0.06	0.77	0.25	0.37
**Galactose**	**0.00**	**0.00**	0.98	0.29	0.68	0.92	0.63
**Gluconic acid**	**0.00**	**0.02**	0.31	0.31	0.51	0.34	0.22
**Hexose**	**0.00**	0.09	0.83	0.20	0.87	0.12	0.12
**Lactic acid**	**0.00**	**0.00**	0.84	0.31	0.34	0.23	0.19
**Fructose**	**0.00**	**0.00**	0.85	0.48	0.94	0.39	0.79
**Raffinose**	**0.00**	**0.00**	0.69	**0.00**	0.77	0.59	0.33
**Sophorose**	**0.00**	0.56	0.82	0.69	0.92	0.98	0.13
**Sucrose**	**0.00**	**0.00**	0.28	0.39	0.98	0.11	0.81
**Tagatose**	**0.00**	**0.00**	0.98	0.34	0.87	0.82	0.62
**Erythritol**	**0.00**	0.12	0.69	**0.04**	0.67	0.46	0.60
**Methyl O-D-galactopyranoside**	0.12	**0.05**	0.50	**0.00**	0.89	0.32	0.86
**Glyceric acid**	**0.01**	**0.02**	0.13	0.25	0.90	0.45	0.32
**Pantothenic acid**	**0.00**	**0.00**	0.24	0.27	0.95	0.75	0.58
**Ascorbic acid**	0.51	0.77	**0.04**	**0.01**	0.36	0.06	0.37
**Dehydroascorbic acid**	0.94	0.31	**0.00**	**0.00**	0.06	**0.01**	**0.03**
**Isothreonic acid**	0.17	**0.00**	0.34	0.99	0.06	0.18	**0.03**
**Mannonic acid**	0.77	**0.00**	0.28	0.25	0.26	0.14	0.58
**Threonic acid**	**0.05**	**0.00**	**0.01**	**0.00**	0.40	0.08	0.08
**α-tocopherol**	**0.00**	0.84	**0.01**	0.46	0.07	0.37	0.39
**Citric acid**	**0.00**	0.78	**0.03**	**0.01**	0.91	0.60	0.43
**Succinic acid**	0.35	**0.00**	**0.01**	**0.01**	0.71	**0.02**	0.12
**3,4-Dihydroxycinnamic acid**	**0.00**	0.68	0.44	**0.04**	0.32	0.93	0.82
**cis-Caffeic acid**	**0.00**	0.61	0.26	**0.00**	0.66	0.89	0.92
**Shikimic acid**	**0.00**	**0.00**	0.46	0.06	0.50	0.70	0.21
**5-Hydroxynorvaline**	**0.00**	0.50	0.17	0.80	0.70	0.37	0.86
**Asparagine**	**0.00**	**0.00**	0.47	0.13	0.62	0.36	0.15
**Aspartic acid**	0.31	**0.04**	**0.01**	0.74	0.58	0.94	0.56
**Phenylalanine**	**0.00**	**0.03**	0.54	0.56	**0.01**	0.29	0.41
**Phenylethylamine**	0.40	**0.00**	0.68	0.42	0.39	0.49	0.51
**Serine**	**0.00**	**0.02**	0.69	0.28	**0.04**	0.52	0.59
**Threonine**	**0.00**	**0.04**	0.52	0.83	0.27	0.31	0.09
**Tryptophan**	**0.00**	**0.00**	0.30	0.50	0.10	0.56	0.23
**Tyrosine**	**0.00**	**0.01**	**0.03**	0.98	0.05	0.68	0.08
**Methionine sulfoxide**	0.74	**0.00**	**0.01**	0.60	0.17	0.45	**0.00**
**Citrulline**	0.10	**0.00**	0.47	0.30	0.55	0.68	0.59
**Ornithine**	**0.01**	**0.00**	0.70	0.13	0.15	0.78	0.75
**Spermidine**	**0.00**	0.20	0.15	0.48	0.21	0.66	0.45
**Urea**	0.11	**0.00**	**0.02**	0.68	0.30	0.06	0.52
**Allantoic acid**	**0.00**	**0.00**	0.95	0.40	0.47	0.16	0.09
**Orotic acid**	**0.00**	**0.00**	0.27	0.80	0.89	0.97	0.99
**Phosphate**	0.06	**0.02**	0.07	0.26	0.26	0.46	0.14
**Total significant**	30	31	10	10	2	2	3
**Total independent**	22	25	7	9	2	1	3

Significant p-values are in bold and red. The underline shows the significant independent effect that is not included in a significant interaction and the more complex interaction that includes the effect of independent factors or more simple interactions (the posterior interpretation is only based on the complex interactions that nest the simple effect and the independent effect that are not included in an interaction).

Interestingly, from these 30 metabolites that were displayed differentially between ecotypes, 21 were also affected by salinity treatment, namely, glucose, galactose, gluconic acid, lactic acid, fructose, raffinose, sucrose, tagatose, glyceric acid, pantothenic acid, threonic acid, shikimic acid, asparagine, phenylalanine, serine, threonine, tryptophan, tyrosine, ornithine, allantoic acid, and orotic acid. Additionally, seven metabolites were modulated by salinity but not for ecotype, namely, mannonic acid, aspartic acid, phenylethylamine, citrulline, urea, and phosphate (**Table 1**).

Significant E × S independent interactions were found for the following nine metabolites: raffinose, erythritol, methyl O-D-galactopyranoside, ascorbic acid, threonic acid, citric acid, succinic acid, 3,4-dihydroxycinnamic acid, and cis-caffeic acid.

Regarding seed HP, from the 10 that changed significantly, 7 had a significant independent effect, namely, ascorbic acid, threonic acid, alpha-tocopherol, citric acid, aspartic acid, tyrosine, and urea.

Significant E × P interactions were found for phenylalanine (*p ≤*0.01) and serine (*p* ≤ 0.05). Seed HP induced an increase of phenylalanine and serine in BO78, and a decrease of these amino acids in Socaire.

Significant S × P interaction was observed for succinic acid (that also presented S × E interactions), suggesting that the effect of HP on their level is dependent on the treatment but not on the ecotype.

Interactions among three factors (E × S × P) were found for dehydroascorbic acid (the oxidized form of ascorbic acid), isothreonic acid (product of catabolism of ascorbic acid), and methionine sulfoxide (product of methionine oxidation). HP reduces the level of methionine sulfoxide and isothreonic acid under saline conditions in both ecotypes, but at control conditions, the response level contrasted between ecotypes. Additionally, HP increased the level of dehydroascorbic acid under control and salinity in Socaire, but in BO7, this is observed only in saline conditions, reaching a considerably higher level than in the tolerant ecotype. In fact, dehydroascorbic acid and ascorbic acid were the most modified metabolites through the metabolomic profile.

## Discussion

Here, we postulate that seed halo-priming induces a metabolic stress memory that is different from the programming induced by salinity and might be more important for the tolerance of the most sensitive ecotype.

In this study, ecotype was an important factor in explaining the variability of germination capacity under saline conditions (**Figure 1**), the ability to recover *P_n_* after a shock of saline stress (**Figure 2**), and the extent of seed HP effect on germination (**Figure 3**) and during juvenile phase (**Figure 4**). In fact, their metabolites (**Figure 5**) and metabolomic profile showed a clear clade separation between ecotypes (**Figure 6**).

Considering the changing metabolites in the E × S interaction, we suggest that carbohydrates have a central role as a C source for interconnection routes. Salinity maintains TSS (**Figure 5** and **Supplementary Table 1**) but reduces levels of those carbohydrates involved in the glycolysis pathway. In the case of Socaire, this could be related to the increasingly significant level of raffinose, erythritol, and ascorbic acid (**Table 1** and **Figure 6C**). Raffinose and erythritol increase the osmotic potential of the cell, helping to maintain osmotic and redox balance in many species in response to salinity, even in halophytes (Zhang et al., 2018; Jiménez-Becker et al., 2019). Ascorbic acid participates in the stromal AsA-GSH cycle and water–water cycle. The water–water cycle is essential for suppressing the photoinhibition by scavenging of ROS generated in chloroplasts, and by the dissipation of energy as an alternative electron flux (Asada, 2000). On the other hand, BO78, but not Socaire, increases the level of methyl O-D-galactopyranoside, which could be related to limiting the accumulation of methanol and the toxic formaldehyde production in the cytoplasm produced by saline conditions (Gout et al., 2000; Aubert et al., 2004). Alternatively to the role of producing other carbohydrates, the reduction of sugar levels could be related to feeding another important pathway: the shikimate pathway. This relates to the metabolism of carbohydrates with aromatic amino acid production (and their secondary metabolites). Shikimic acid content was higher in BO78 than in Socaire, but both ecotypes tended to decrease the aromatic amino acids (phenylalanine, tryptophan, and tyrosine) under salinity conditions (**Table 1**). Interestingly, an alkaloid derived from phenylalanine, phenylethylamine, increased in both ecotypes under salt stress. This biogenic amine is involved in the major pathway for the synthesis of 2-phenylethanol, an aromatic alcohol, playing a major role in plant defense against herbivores and pathogens (Günther et al., 2019). Also, it is suggested that it can be related to polyamine and ethylene accumulation under stress (Irsfeld et al., 2013). Other derivatives from the shikimic acid pathway affected by E × S interactions were 3,4-dihydroxycinnamic acid and cis-caffeic acid. These are important polyphenols with osmoregulatory and high antioxidant capacity (Nishizawa et al., 2008) and are candidates to be part of the salt tolerance mechanism of Socaire but not for BO78 (**Figure 6** and **Table 1**). We suggest that the induction of raffinose, erythritol, ascorbic acid, 3,4-dihydroxycinnamic acid, and cis-caffeic acid could be key molecules that explain the lower level of lipid oxidation (measured as MDA) under saline conditions in Socaire compared to BO78 (**Figure 4**; **Supplementary Table 1**).

Citric and succinic acids are two main components of the TCA cycle that are maintained in the tolerant ecotype and decreased in the sensitive ecotype. It is known that the TCA cycle can influence the carbon balance and partition to growth, supplying electrons (succinate) to the ETC (electron transport chain in the mitochondria) and directly synthesizing compatible solutes such as sugars and amino acids and reactive oxygen species (ROS) detoxification (Che-Othman et al., 2017). The TCA network is flexible under salinity stress to enable to fulfill cellular energy demands, including the induction of the malate/pyruvate pathway (Kazachkova et al., 2013) and the γ-aminobutyric acid (GABA) shunt (Che-Othman et al., 2020).

The majority of the N-related metabolites changing were downregulated by salinity treatments, except by the following amino acids: proline, serine, threonine, and aspartic acid. Interestingly, the increased level of these amino acids was similarly reported for *Sargassum muticum* and *Jania rubens*, attributed to an osmopotential role (Abdel Latef et al., 2017). Here, we suggest that serine and aspartic acid can feed the TCA cycle through its conversion into pyruvate and oxaloacetate (or fumarate), respectively. Moreover, serine increase could be associated with photorespiratory reactions that can dissipate excess reducing equivalents and energy, providing an internal CO_2_ pool and NADH used for respiration in the mitochondria (Busch, 2020). Additionally, proline was positively regulated under salt and is considered an important osmoregulator involved in the transport of NaCl into the bladder cells in quinoa salt-tolerant ecotypes (Böhm et al., 2018). Indeed, it has been pointed out as one major factor in the regulation of the expulsion of NaCl crystals in leaves of quinoa (Böhm et al., 2018).

It is noteworthy that while salinity affects C- and N-related metabolites, we found no interactions between E × S in N-content metabolites. In fact, salinity affects similarly N-related compounds independent of the constitutive tolerance level of the ecotypes. This response could be related to the great disruption that saline involves in the acquisition of macronutrients including N and P (Miranda-Apodaca et al., 2020; Bouras et al., 2022).

The metabolic basis of plant stress memory and how these affect the physiology of plants are still largely unknown. Moreno et al. (2018) reported that seed hydro-priming and osmo-priming enhanced germination-related traits of Titicaca cultivar, a hybrid variety of quinoa breeds from a Peruvian and a southern Chilean landrace. Our results indicate that, under saline conditions, seed HP affected the germination of the most saline-sensitive ecotype, BO78, allowing an increase in both the speed of germination and final germination percent (**Figure 3**). By contrast, seed HP did not affect the germination parameters of the Altiplano ecotype (Socaire) under saline conditions (**Figure 3**). Thus, we found that seed halo-priming had a differential effect on the germination traits of quinoa depending on the salt tolerance ecotype.

In juvenile plants, the level of the quantum yield of PSII (ΦPSII) in BO78 under saline conditions improved significantly by seed HP (**Figure 4**). This enhancement was concomitant with a reduction process that dissipates as heat the excess of absorbed energy in the PSII antenna (NPQ to antenna level). Our results suggest that seed HP modified the photochemistry of PSII in BO78 towards an increased capacity to use the light energy in the photochemical process, which eventually may improve its photosynthetic performance under salinity conditions. We suggest that the changes in ascorbic acid and α-tocopherol induced by seed HP can act synergistically to preserve plastid redox homeostasis and protect PSII, thus promoting the use of energy in the photochemical process (Szarka et al., 2012). The changes in the levels of dehydroascorbic acid, isothreonic acid, and threonic acid are in concordance with the promotion of ascorbic acid metabolism induced by seed HP. Regarding α-tocopherol, it is known that it can neutralize lipid peroxy-radicals, consequently blocking lipid peroxidation by quenching oxidative cations and then maintaining cell membrane stability (Chen et al., 2018). The increased level of α-tocopherol induced by HP could be related to the maintenance of the integrity and fluidity of photosynthesizing membranes, contributing to enhanced salt resistance.

We suggest that the increase in ascorbic acid and α-tocopherol by seed HP is sustained in the use of carbohydrates (starch decrease by HP). Recently, it has been reported that Tyr (modulated by P), a derivate of Phe, could be involved in the biosynthesis of α-tocopherol (Tohge et al., 2013). The optimization of the shikimate pathway could be in the dismemberment TCA metabolite (citrate and succinic) with a decrement in amino acid production derivate from TCA (such as Asp), probably to avoid the depletion of carbohydrates under salinity stresses (Hill et al., 2013).

**Figure 7:**
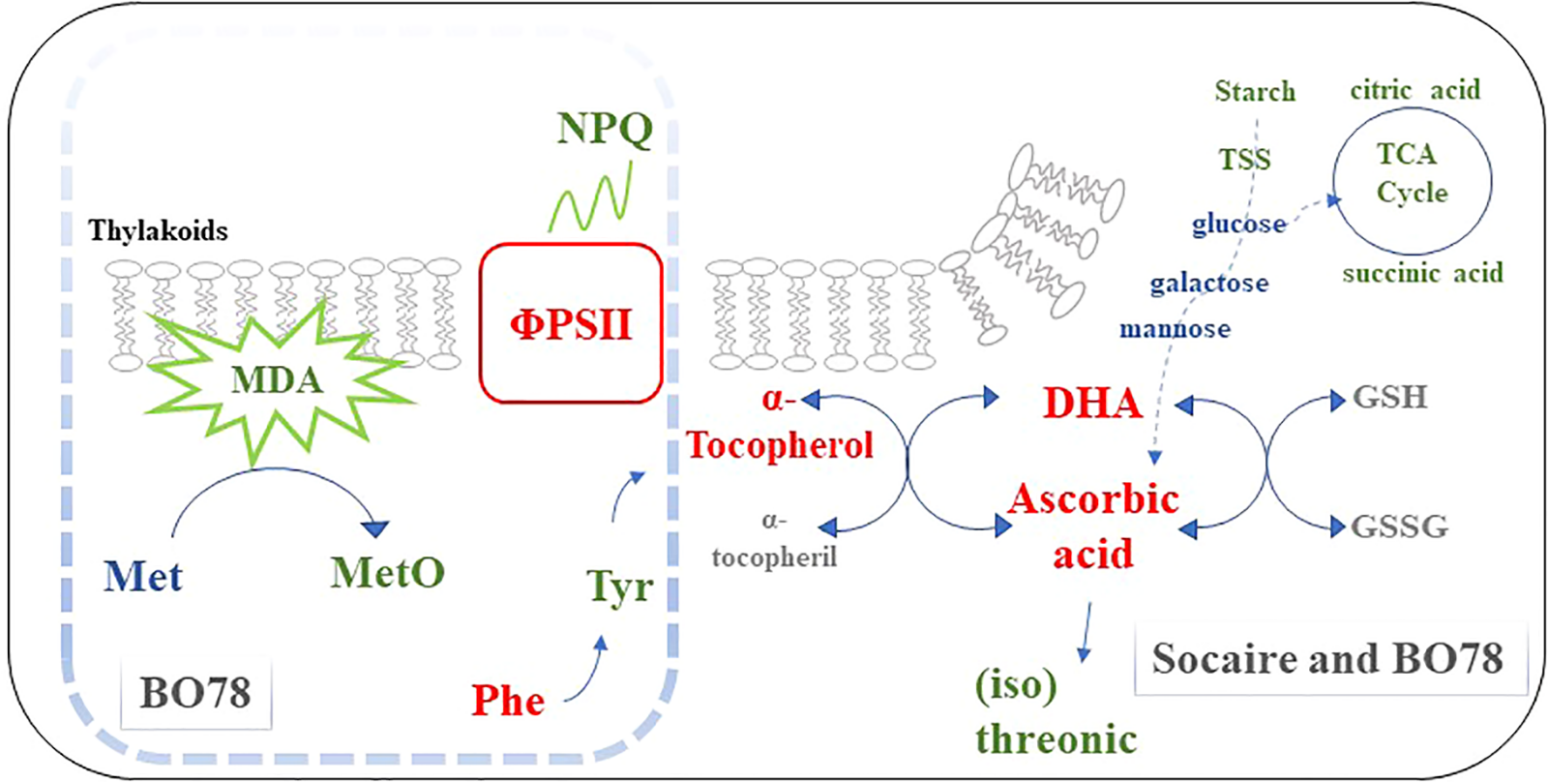
Simplified schematic of seed HP effect on physiological processes and metabolites in *C. quinoa* ecotypes. Seed HP (as a unique factor) induces a reduction of starch and citric acid, and, in interaction with salinity, reduced TSS and succinic acid; however, seed HP maintains glucose, galactose, and mannose, which are important substrates for ascorbic acid biosynthesis. Seed HP enhanced the level of ascorbic acid, dehydroascorbic (DHA), and α-tocopherol, which are involved in antioxidant cycles associated with stabilize membranes. Additionally, seed HP reduced levels of threonic and isothreonic acids, suggesting that ascorbic acid could be less degraded. It is suggested that the higher level of α-tocopherol contributes to this response. The enhanced aromatic amino acids pathways (Phe and Tyr) observed in BO78 have been pointed out as an alternative pathway to produce α-tocopherol. Finally, the level of oxidative markers (methionine sulfoxide and MDA) (that increases under saline conditions) was reduced by the effect of seed HP, and further reductions were observed in the most sensitive than in the tolerant ecotype. We suggest that these changes might increase the robustness of PSII through an increase of ΦPSII and a decrease of NPQ, which was observed in the most sensitive ecotype (BO78). Red, green, and blue colors represent an increase, reduction, or non-change in the physiological process or metabolite accumulation upon HP (compared to UP) or HP-S (compared to UP-S treatment), respectively. The gray color represents metabolites that were not present (or not identified) in the metabolome profiling. The processes and metabolites inside the square dotted line correspond to changes only in the BO78 ecotype.

The increase in ascorbic acid and α-tocopherol induced by seed HP affected the redox status more in the sensitive ecotype than in the tolerant one. This is observed in lipid peroxidation (measured as MDA levels) and methionine sulfoxide (product of protein oxidation), which were further reduced in the sensitive ecotype during salinity. Considering that chloroplast is one of the most important sources of plant ROS, we postulate that enhancing ΦPSII and decreasing NPQ in the most sensitive ecotype could be the result of changes in oxidative status. A schematic representation of the effect of HP on metabolites and physiology is presented in **Figure 7**.

There is also emerging evidence that redox status, promoted by priming, can impact chromatin methylation and epigenetics (Locato et al., 2018). For instance, ascorbic acid may act as a cofactor for dioxygenases that modify chromatin and DNA (Reid et al., 2017). Contrastingly, succinate can inhibit the activity of chromatin-modifying enzymes. Thus, it seems that metabolic routes involving ascorbic acid and succinic acid could lead to a higher level of demethylation and changes in gene expression under saline conditions. The crossroads of the metabolism and the mechanisms controlling chromatin methylation and epigenetics need to be tested in plants with contrasting tolerance.

## Conclusions

In summary, Altiplano ecotypes of quinoa could be more tolerant than the Lowland ecotype because of the constitutive high storage of osmolytes and antioxidants that are also induced by salinity. Our work supports the idea that salinity differentially compromises the C between ecotypes, while N metabolism was strongly affected in both. Contrastingly, seed HP finely orchestrated changes in relation to the antioxidant metabolism related to ascorbic acid and α-tocopherol, inducing a metabolic imprint that has effects during germination and plant growth. The differential signature of seed HP in ecotypes confers improved tolerance to salinity to the most sensitive ecotype, which is observed in the strengthening of PSII. Future investigations must study the reciprocal role of a metabolic imprint and the epigenetic response.

## Data availability statement

The datasets presented in this study can be found in online repositories. The names of the repository/repositories and accession number(s) can be found in the article/**Supplementary Material**.

## Author contributions

LB-G, LC, NF, EO-G, SF, and PC designed the assays and led the writing of the manuscript. MG conducted metabolomics analysis. RÁ and KP grew both C. quinoa genotypes. CS, GB, JO, and CC performed the assays and measurements. LB-G, ND-S, and PC led the supported projects and edited the manuscript. All authors read, edited and approved the final version of the manuscript for publication.

## Supplementary material

Supplementary Figure 1 Effect of different seed priming treatments on germination percentage in BO78 ecotype of quinoa. Seeds were primed with various treatments and then germinated at 300 mM of NaCl (Details in Materials and Methods). Error bars show mean ± SE (n=50).

Supplementary Table 1 Effect of seed HP on metabolites and biomass under Control or Salinity conditions in two genotypes of *C. quinoa*. Different parameters were determined in four different individuals (*n* = 4). Fully expanded third leaves (from the top part of the plant) were used for measurements. Statistical differences were established (P < 0.05) using three-way ANOVA. The last rows of the table show the significance levels (P) of the factors (E, S and P) and interactions. Significant P-values are in bold and red. The underline shows the significant independent effect that are not included in a significant interaction, and the more complex interaction that includes the effect of independent factors or more simple interactions (The posterior interpretation is only based on the complex interactions that nests the simple effect, and independent effect that are not included in an interaction).

Dataset 1 The file shows the metabolome data obtained from the West Coast Metabolomics Center (UC Davis, Davis, CA, USA) using leaves of juvenile plants of two ecotypes of Quinoa subjected to seed priming (UP or HP) and grown under Control or saline stress conditions.
