# Supplementary figures and images for: Metabolic imprint induced by seed halo-priming promotes a differential physiological performance in two contrasting quinoa ecotypes
Source: Front Plant Sci. 2023 Feb 14;13:1034788. doi: 10.3389/fpls.2022.1034788 (PMC9971973; doi:10.3389/fpls.2022.1034788)

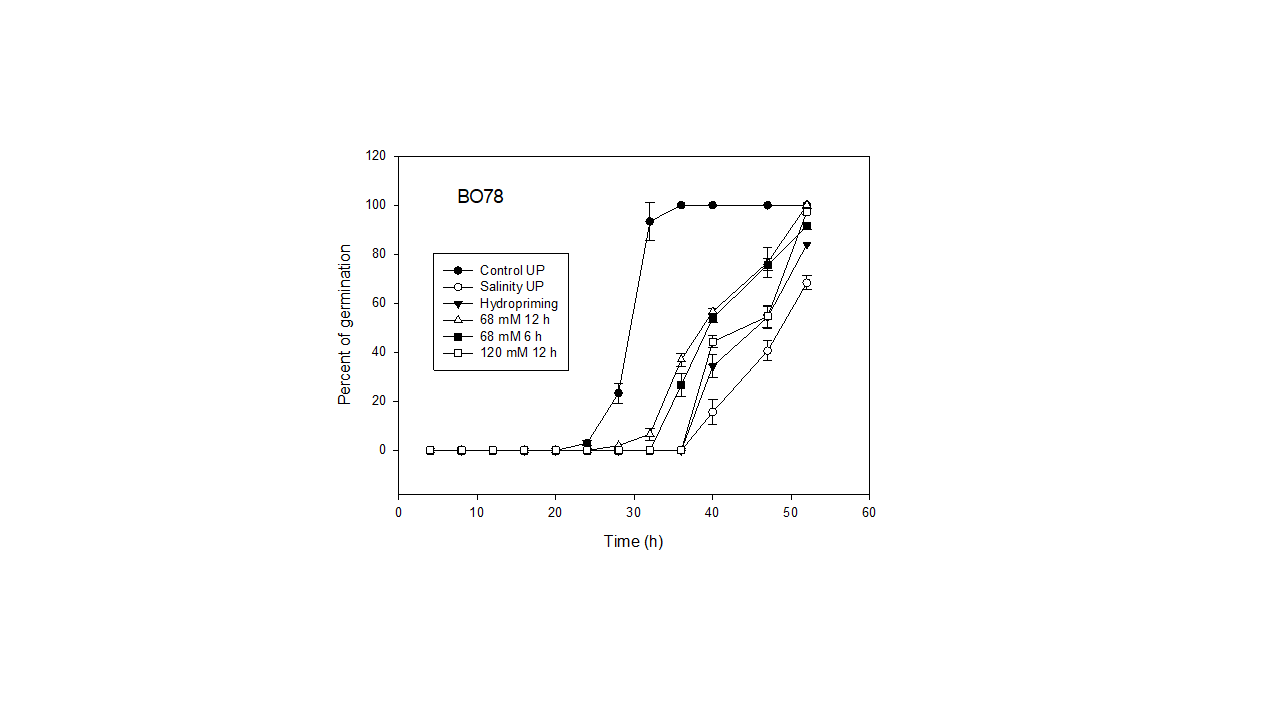

Supplement: Supplementary Figure 1 — Effect of different seed priming treatments on germination percentage in BO78 ecotype of quinoa. Seeds were primed with various treatments and then germinated at 300 mM of NaCl (Details in Materials and Methods). Error bars show mean ± SE (n=50). [file Image_1.tif]
