# Supplementary material for: Metabolic imprint induced by seed halo-priming promotes a differential physiological performance in two contrasting quinoa ecotypes
Source: Front Plant Sci. 2023 Feb 14;13:1034788. doi: 10.3389/fpls.2022.1034788 (PMC9971973; doi:10.3389/fpls.2022.1034788)
Supplement: Supplementary Table 1 — Effect of seed HP on metabolites and biomass under Control or Salinity conditions in two genotypes of C. quinoa. Different parameters were determined in four different individuals (n = 4). Fully expanded third leaves (from the top part of the plant) were used for measurements. Statistical differences were established (P < 0.05) using three-way ANOVA. The last rows of the table show the significance levels (P) of the factors (E, S and P) and interactions. Significant P-values are in bold and red. The underline shows the significant independent effect that are not included in a significant interaction, and the more complex interaction that includes the effect of independent factors or more simple interactions (The posterior interpretation is only based on the complex interactions that nests the simple effect, and independent effect that are not included in an interaction). [file Table_1.docx]

| E | P | S | TTS  mg gFW^-1^ | Starch  mg gFW^-1^ | Chlorophyll *a*  mg g FW^-1^ | Chlorophyll *b*  mg g FW^-1^ | Proline  mg g FW^-1^ | MDA  nM gFW-^1^ |
| --- | --- | --- | --- | --- | --- | --- | --- | --- |
| Socaire | UP | C | 20.68 ± 0.17 | 38.07 ± 2.90 | 1.87 ± 0.08 | 0.65 ± 0.02 | 0.69 ± 0.08 | 2.81 ± 0.04 |
| Socaire | HP | C | 18.58 ± 0.46 | 30.08 ± 1.39 | 2.28 ± 0.05 | 0.80 ± 0.02 | 0.53 ± 0.05 | 2.57 ± 0.18 |
| Socaire | UP | S | 23.74 ± 0.71 | 37.88 ± 3.17 | 2.14 ± 0.09 | 0.76 ± 0.05 | 1.13 ± 0.09 | 5.52 ± 0.28 |
| Socaire | HP | S | 19.49 ± 0.53 | 27.83 ± 1.65 | 2.22 ± 0.06 | 0.87 ± 0.05 | 0.52 ± 0.05 | 2.55 ± 0.19 |
| BO78 | UP | C | 22.76 ± 0.77 | 45.16 ± 2.00 | 3.57 ± 0.14 | 1.17 ± 0.05 | 0.79 ± 0.05 | 3.67 ± 0.17 |
| BO78 | HP | C | 18.79 ± 0.64 | 41.40 ± 3.04 | 3.16 ± 0.27 | 1.07 ± 0.10 | 0.67 ± 0.12 | 3.51 ± 0.15 |
| BO78 | UP | S | 21.06 ± 0.68 | 42.67 ± 1.60 | 3.51 ± 0.09 | 1.25 ± 0.04 | 1.18 ± 0.20 | 8.02 ±0.33 |
| BO78 | HP | S | 20.10 ± 0.51 | 37.21 ± 1.64 | 3.31 ± 0.19 | 1.20 ± 0.07 | 0.68 ± 0.07 | 3.50 ± 0.14 |
| E | | | 0.897 | **<0.001** | **<0.001** | **<0.001** | 0.12 | **<0.001** |
| P | | | **<0.001** | **<0.001** | 0.752 | 0.505 | **<0.001** | **<0.001** |
| S | | | **0.041** | 0.171 | 0.468 | **0.018** | 0.008 | **<0.001** |
| E x P | | | 0.401 | 0.184 | **0.012** | **0.013** | 0.603 | 0.018 |
| E x S | | | **0.015** | 0.516 | 0.774 | 0.814 | 0.924 | 0.009 |
| S x P | | | 0.608 | 0.566 | 0.774 | 0.903 | **0.008** | **<0.001** |
| E x S x P | | | **0.005** | 0.956 | 0.189 | 0.603 | 0.808 | **0.009** |

**Supplementary Table S1.** Effect of seed HP on metabolites and biomass under Control or Salinity conditions in two genotypes of C. quinoa. Different parameters were determined in four different individuals (n = 4). Fully expanded third leaves (from the top part of the plant) were used for measurements. Statistical differences were established (P < 0.05) using three-way ANOVA. The last rows of the table show the significance levels (P) of the factors (E, S and P) and interactions. Significant P-values are in bold and red. The underline shows the significant independent effect that are not included in a significant interaction, and the more complex interaction that includes the effect of independent factors or more simple interactions (The posterior interpretation is only based on the complex interactions that nests the simple effect, and independent effect that are not included in an interaction).
